# Experiences of individuals with intellectual disability who lecture in higher education

**DOI:** 10.3389/fpsyt.2023.1258337

**Published:** 2023-11-22

**Authors:** Silje Mevold, Leif Inge Johansen, Rolf Wynn, Gro H. Ramsdal

**Affiliations:** ^1^Department of Social Education, Faculty of Health Sciences, UiT The Arctic University of Norway, Tromsø, Norway; ^2^Department of Clinical Medicine, Faculty of Health Sciences, UiT The Arctic University of Norway, Tromsø, Norway; ^3^Department of Education, ICT and Learning, Østfold University College, Halden, Norway

**Keywords:** intellectual disability, life satisfaction, inclusion, autonomy, work

## Abstract

**Introduction:**

Prior studies have suggested that adults with intellectual disabilities who are in employment in general report a high level of well-being and life satisfaction. Less is known about which experiences and outcomes that are most important for the experiences of those who are employed.

**Methods:**

We interviewed six persons with intellectual disabilities that worked as lecturers at a health and social education programme at a university about which experiences and outcomes that they believed were of importance to their work experiences.

**Results:**

The participants spontaneously focused on three main themes that described their experiences with work: the high degree of autonomy at work, their involvement in positive new relationships, and their experiences of accomplishment and coping on new social arenas. Although there were challenges, these were often overcome by working closely with another teacher.

**Discussion:**

The three main themes by which the participants described their work experiences and outcomes can be related to the concepts of autonomy, relatedness, and competence. The experiences described by the participants are valuable for the future facilitation of work assignments for people with intellectual disability in ordinary working life.

## Introduction

People with intellectual disabilities are underrepresented in the work force (1, 2). Current estimates indicate that the employment rate for people with intellectual disabilities varies between 9 and 40% across different countries (1), and little change has been found over time in rates of integrated employment in adults with intellectual disabilities (3). The exact employment rate in Norway is not known, but a much-cited estimate indicates that one out of four individuals with intellectual disabilities has some kind of employment, mostly in sheltered employment (4).

These estimates present an important challenge in the struggle to include individuals with intellectual disabilities in society, since research repeatedly has demonstrated the importance of work to people in general (5), and specifically when it comes to the social and financial involvement of persons with disabilities (6). Adults with intellectual disabilities reported that work was one out of two factors contributing most to their life satisfaction (7). Individuals with intellectual disabilities in open employment also showed significantly higher quality of life scores (8). Reviewing the literature, Jahoda et al. (9) reported consistently *higher well-being and quality of life* in individuals with intellectual disabilities in supported employment.

However, most studies have not asked individuals with intellectual disabilities themselves about their work experiences (10). Furthermore, according to Dean et al. (11, p. 2) ‘the benefits of employment and employment support are predominantly discussed in terms of *access* to employment’. Most studies in employment support have centred on skills and abilities (12). A scoping review concluded that most of the employment-related research in intellectual disability from 2000 to 2010 focused on identifying the work role achieved like employment access, payrates and job titles (6). Fewer than five articles in this review investigated central aspects of inclusion like the *interaction processes* involved in developing a sense of belonging, reciprocity and need fulfilment.

Studying the interaction between the needs of people with intellectual disabilities and their working environment, and their experiences with these interaction processes, might be carried out in various work environments. One environment of specific interest is higher education. The principle of participation has become significant in the education of health and social care workers, although the extent of such participation and how people with intellectual disabilities will participate is less clear (13). Inviting representatives of a particular group of service users to share their life stories, is one method of inclusion described in research literature (14). These studies mainly focused on how students and teachers experienced the involvement of service users as lecturers. We found it difficult to locate any studies focusing *the experiences of individuals with intellectual disabilities* employed as lecturers in higher education. Little is known about how these lecturers experience their work assignments with telling their life stories and being experts on their own lives. The absence of such literature seems to indicate a significant knowledge gap. To facilitate more involvement in working life, there is a need for a broad-spectrum description of various types of work experiences from individuals with intellectual disabilities. For them, employment in higher education can be especially helpful, due to the potential of such a high-status work arena in reducing stigma.

Summing up, the statistics show that individuals with intellectual disabilities participate much less in working life than other people (1, 4) in spite of the fact that several studies have reported crucial benefits of such participation (6, 7, 9). Research on the work participation of individuals with intellectual disabilities has only infrequently focused on their own experiences of need satisfaction in interaction with various working environments. Having worked in a college employing individuals with intellectual disabilities as lecturers, we saw an opportunity to learn from them about their experiences working in higher education. These lecturers are hired on an equal footing with other external lecturers and receive regular pay. Another teacher may be present in the classroom to be called on if the lecturer expressively wants this. These experts on their own lives have mainly contributed to ordinary classroom teaching. The purpose of this work inclusion has been multi-faceted, but the idea of letting students hear the service recipients’ own stories about living with a cognitive impairment has played a key role. These work assignments are short-lived: only 2 h once or twice a year, but are still an opportunity for participants to explore experiences with an ordinary working life situation. We wanted people with intellectual disabilities themselves to describe their experiences with these work assignments and the outcomes related to their need fulfilment and well-being. As higher education is a working arena employing very few individuals with intellectual disabilities, this inquiry became especially interesting. Thus, we formulated the following research question:

*What experiences does a group of lecturers with intellectual disabilities have when exploring work assignments as lecturers at a health and social education programme, and what outcomes do they report related to these exploration processes?*

## Methods

### Methodological approach

The researchers followed the general research ethics guidelines from the National Research Ethics Committees in the process of recruitment, consent, data collection and publication (15). The study was approved by the Norwegian Centre for Research Data (NSD project no. 454008). The participants gave both their oral and written consent to participate in the study. The study was based on qualitative interview data involving six adult participants with cognitive impairments who talked about their experiences regarding work assignments as lecturers in a health and social education programme.

Although the communicative challenges in this study constituted a vulnerability, we are of the same opinion as Sigstad and Garrels (16) in that there are several good reasons for including people with intellectual disabilities in research. If we are to gain knowledge about this group’s work life experiences, we must ask those involved. If vulnerable groups are not included in research, there is a risk of violating the Convention on the Rights of Persons with Disabilities (CRPD) principle of equal treatment (17). Furthermore, it is equally important that people with intellectual disabilities seem to appreciate being given the opportunity for this type of participation and inclusion (16).

### Recruitment

The participants in the study were recruited from a list of lecturers on a health and social education programme in Norway. The only inclusion criterion was being an individual with intellectual disabilities and being on the lecturer list of this education program with the work assignment to teach about their own experiences of living with light or moderate cognitive impairment. All the lecturers with intellectual disabilities on the list were included in the recruitment process and they all consented to participate. Potential participants were asked if they would like to participate via a mobile phone text message. This request was followed up by a subsequent telephone conversation in which the purpose of the study and the procedure for a possible interview were explained in detail. It was stressed that participation was voluntary and that not wanting to participate would have no consequences for them or their job assignment at the workplace. During the first meeting, the participants received an information sheet that was thoroughly explained to them. Here, important prerequisites such as voluntary participation and anonymity were emphasized. In this study, there was an increased risk of being recognized because the names of lecturers at educational institutions are publicly available and because this group is relatively small. The participants were therefore made aware that even though we did everything we could to anonymize the data, there was still a certain risk that someone might recognize them. This aspect of the study was also discussed thoroughly and repeatedly with NSD.

### The participants

The participant group consisted of three women and three men, between 30 and 50 years old, who had cognitive impairment. All had capacity to consent. The majority had other jobs within both Permanently Adapted Work (PAW) and Permanently Adapted Work in Ordinary Companies (PAWO), which constituted a significant part of their working day. All participants had employment contracts as lecturers. The duration of the lectures given at the health and social education program were from 1 to 2 h once or twice a year. Length of service as a lecturer at the university ranged from 1 year up to 8 years.

### The interviews

In this study, qualitative semi-structured interviews were chosen. The questions in the interview guide were open-ended and allowed for follow-up questions. Questions tapped the participants’ experiences with lecturing in higher education and in particular the outcomes of their learning processes related to teaching at the university. Main topics were first ‘Work inclusion experiences’ containing questions like: ‘Can you tell me a bit about the job you are doing here?’, ‘How do you like this job?’, ‘How do you like lecturing in front of the students?’, ‘What is good about this job?’, ‘What is not so good about this job?’; second ‘Equality experiences’ including questions such as ‘How are you treated by your colleagues at the university?’, ‘Do you think that your lectures are appreciated?’, ‘Do you get to decide what to say in your lectures?’; and finally ‘Communicative experiences’ including questions such as ‘What do you want to teach the students?’, ‘How did the students respond to your story?’, and ‘Who did you talk to during the breaks?’

All interviews were conducted in the spring of 2022 at the relevant workplace because this was familiar to the informants. The interviews lasted between 50 and 70 min. The interviewer tried to adapt the interviews to the individual participant’s needs in terms of communicative clarity, time span, breaks, etc. We focused on creating a relaxed and caring atmosphere. Such preparation is crucial in all research interviews but is of particular importance for participants with cognitive impairments. This is because persons with intellectual disabilities may experience challenges related to language comprehension, working memory, taking turns, and challenges in terms of the time it takes to process new information (18, 19). Sigstad and Garrels (16) focus on three communication techniques that they believe are suitable for helping participants with this type of disability to give rich descriptions of their experiences and viewpoints. The three techniques are as follows: (1) Demonstrate an expectant and encouraging attitude that tolerates silence. (2) Formulate simple questions and preferably rephrase them so that the questions are more specific and concrete. (3) Repeat, summarize and rephrase the participant’s answers to ensure that the participant’s answers have been understood in the way the answer was intended. The interviewer, who has extensive experience from environmental therapy work involving people with intellectual disabilities, focused on following this communicative advice in all the interviews.

Unfortunately, we were unable to include individuals with intellectual disabilities in the research team, because the individuals with the relevant experience were all included in the planned research sample. The accessible sample of 6 was small, and reducing this sample might further compromise the theoretical saturation (20). Other potential co-researchers with relevant experience lived on the other side of the country. Accordingly, geography and the requirements of research ethics made it very difficult to establish the prescribed type of relationships required to implement this type of inclusive research.

### Data analysis

All interviews were audio recorded and transcribed. The interviews were analyzed using the method of thematic analysis (21). The process of analysis was inspired by Malterud’s systematic text condensation method (22–24). This is a cross-sectional analysis used to summarize and interpret qualitative data. The analysis consists of four steps. In step one, it is important for the researcher to ‘listen to the voice of the informants’. This kind of openness challenges the researchers to ignore personal prejudices and avoid interpreting data according to their own theoretical orientation. The researchers in our study repeatedly read the interviews separately trying to ‘listen to the voice of the informant’, as recommended by Malterud (22–24), and to familiarize themselves with the raw text on their own. Then the researchers came together, sharing thoughts and preliminary themes. Although the wording of themes varied somewhat among the researchers, their choice of content and interpretations of main topics in the interviews were mostly coinciding. Interviews often suggest many themes and not all can be covered in one analysis. Thus, this early process was aimed at forming an overview and identifying interesting themes emerging in several or most of the interviews, themes like ‘experiencing the teacher role’, ‘self-regulation in class’ and ‘experiences of empowerment’.

The second step in systematic text condensation focuses on moving from themes to codes, sorting, finding and marking relevant text in a systematic review and organization of the data material (22–24). Working together and individually, the researchers in our study started the process of highlighting sections of our text, phrases or sentences of relevance and finding codes to describe their content, but also looking for relationships between different codes. Codes like ‘validation experiences in class’ and ‘support from other teachers’ and ‘connecting during breaks’ were found to be related.

The third step in this analyzing process involves condensing the material into code groups by focusing on words or expressions that the informants have used in the interviews (22). Looking closely at and comparing the codes, the researchers gradually started to recognize patterns and the codes were organized into groups, thus condensing the data and constituting main themes. This was first done individually and then the researchers looked for common interpretations seeking to formulate theme names that integrated the individually worded codes. For example, codes like ‘connecting during breaks’, ‘validation experiences in class’, ‘support from other teachers’ and ‘meeting outside the university’ were grouped into a main theme, called ‘Experiences of building and maintaining social relations’. Codes including ‘learning to make a PowerPoint presentation’, ‘learning to lecture in front of a group’ and ‘coping with performance pressure’, were grouped into the main theme of ‘Learning and coping experiences’. Finally, ‘freedom to define your own Life story’ and ‘becoming a spokesperson’ constituted a theme called ‘Experiences regarding self-determination’. At this stage, we also went back to the text to compare our main themes to the data to consolidate our themes and see if we were missing something. The main themes were named (1) Experiences of building and maintaining social relations, (2) Learning and coping experiences, and (3) Experiences regarding self-determination.

In the condensation process various ‘code memos’ were used in accordance with Gibbs (25) to explore and develop higher order categories or themes. In step four of the analytical process, the researcher is required to move from condensation to contextualization, by drawing on concepts, theories, and prior research (22). The researchers contextualized the emerging themes by reading up on and involving concepts and theories that could help us understand our data and relate them to prior research. It was during this stage of the process that we discovered how much the data and the emerging themes addressed the idea of need fulfilment of individuals with intellectual disabilities in working life, and we realized that our three main themes were closely related to the three basic needs of relatedness, competence, and autonomy, as theorized and researched by Deci and Ryan (26).

## Results

We present the results under three main headings that refer to the three main themes emerging in our data. Sub-themes are marked with quotation marks to clarify the codes and ideas included in the construction of the three main themes.

### Experiences of establishing and maintaining social relations

This main theme evolved around sub-themes describing the experiences of ‘getting to know the staff’ and ‘the relational challenges’ and ‘vulnerabilities in class’, incorporating problems such as ‘concerns with being ridiculed’ and facing ‘uncomfortable questions’. Overcoming these challenges was much due to experiences of ‘social inclusion’ and ‘support from other teachers’ involving ‘emotional support’, help in ‘connecting during breaks’ and social invitations including ‘meeting outside the university’.

The informants’ descriptions of work at the university often started with their reflections on the experiences they had the first time they lectured. One of the informants described it as fun right from the start, but most of the participants described it as a little scary at first. Consequently, almost all the informants quite spontaneously described the importance of meeting with the university staff as they arrived at campus. They reported feeling welcome and secure when shown into the classroom by someone who knew the students and the facilities, and they stressed the importance of ‘getting to know the staff’ and establish positive relationships.

Time had passed since these first-time experiences and at the time of the interviews, most of the informants had become more experienced lecturers. Some had approximately 3 years of experience, and others had up to 9 years. Regardless of the amount of experience as lecturers, the participants said that they liked working as a lecturer and stated that it was especially nice to meet new people, like students and staff. They said things like:

*The students were great!*

*I liked when the students came to talk to me before the lecture.*

*The teacher from university is very including.*

Although it did feel nice to meet these new people, some informants also experienced a certain tension when faced with unfamiliar faces in a new situation. The biggest ‘relational challenge’ was meeting the students in class. Several informants expressed satisfaction that the students were informed beforehand that the lecturers had an intellectual disability. One participant recalled her concern about encountering students who were mostly in their early twenties wondering if the students were mature enough to relate to her life story. Another informant explicitly described being worried about whether the students would comply with their duty of confidentiality and could be trusted:

... *And then I thought what if they are those kind of teens …. and if I say something, and they do not know what confidentiality is, and then they will just sit there and grin and giggle.*

Other challenges in establishing a relationship with the students concerned trust issues related to their ‘concern about being ridiculed’ or how to cope when encountering certain types of ‘uncomfortable questions’ in front of a group of people. The content of the questions they were concerned about varied. Some worried about theoretical questions or other questions that might be difficult to understand or to which they did not know the answer. As one informant said:

*If they start asking stuff about social education, I do not know what to answer.*

Many participants described the discomfort and anxiety associated with having to face questions they perceived as too personal and invasive, indicating that finding a comfortable level of intimacy in these new relationships were challenging. What was perceived as being *too* private, varied among the participants; for some it was about cohabitation, for others it was about relationships with parents, appearance, or problems at work. One informant said it like this:

*There have been some questions about me and my partner, and things that have to do with our relationship. I do not feel that has anything to do with the public.*

One participant said that relating to students in class sometimes became more challenging due to unexpected events like familiar faces turning up in the crowd. Another participant described her experience of anxiety because a former acquaintance who was now a student sat in the classroom and might start asking about certain things regarding their time at school together. Almost all participants touched on these types of sub-themes related to building and maintaining relationships with students in class. We eventually merged these codes into a sub-theme theme called ‘relational vulnerability in class’, including challenging experiences with exposing their everyday lives with disabilities in front of a group of young people.

Accordingly, the ‘support from other teachers’ proved important in the long term in the sense that with another teacher present the participants felt secure enough in class to come back and lecture another time. The challenges described above made it important to have a support person present in the classroom as a reassurance that the situation would not get out of control for them and to give a helping hand in establishing a comfortable level of intimacy. Although the present teachers were passive listeners most of the time, they represented an ‘emotional support’ that enabled the lecturers to cope with relational challenges in the classroom. This kind of support helped the lecturers stay long enough to find solutions to these relational challenges. Gradually, some participants found ways to pre-empt the questions they feared and had found strategies to avoid difficult questions. One participant described her discovery of how to set clear boundaries from the start of the relationship:

*Because I let them know from day one that those academic terms and things like that, just do not bother!*

Having another teacher present was described as essential for building and maintaining relationships with the students. The informants described how people from the staff were supportive in establishing social contact with students outside the classroom and facilitating ‘connecting during breaks’ and conversations before and after the lecture. Thus, the participants got the chance to experience that students were authentically interested in what they had to say, and the participants got to feel respected and cared for in their new relationships. The informants described their lecturing work as being meaningful and felt that they were valued in this role by the other teachers, by students, but also by people outside the university like friends, acquaintances, and family. Or, as one informant said:

*I simply feel included, I feel like I am being heard. I feel that I am important.*

Three of the informants described how their job assignments as lecturers had led to new opportunities regarding ‘social inclusion’. As they developed relationships with staff at the university, they received invitations to participate in other forums, such as co-research, theater projects and choirs involving ‘meeting outside the university’. One informant had gained a position in a political body for people with disabilities through his contacts with the staff at the university.

Summing up this main theme, participants described the importance of ‘getting to know the staff’ and how being met and welcomed and guided to the classroom was important in a new and confusing social situation. One of the ‘relational challenges’ was becoming acquainted with the students in class and several ‘relational vulnerabilities’ like dealing with ‘uncomfortable questions’ and the ‘fear of being ridiculed’ had to be overcome. Central factors in overcoming these relational challenges were ‘emotional support’ and ‘support from other teachers’. This type of support helped them establish a suitable level of intimacy in class, making it possible to experience a meaningful work assignment and ‘social inclusion’ in the form of feeling valued and respected in their meeting with the students.

### Learning and coping experiences

The ‘learning and coping experience’ theme developed around various sub-themes describing competence experiences from ‘making a PowerPoint presentation’ and then ‘learning to lecture in front af a group,” to competences originating from ‘overcoming challenges’ like ‘coping with performance pressure’. Their ‘new feelings of competence’ depended partly on feed-back experiences like ‘praise from an employer’, but also on ‘flow-experiences’ in class.

The participants described learning new skills and competences lecturing at university. The most obvious competency involved is ‘learning to lecture in front of a group’ including ‘making a PowerPoint presentation’. Some informants had received help with the manuscript, but said they gradually freed themselves from their manuscript and spoke more freely about their experiences with intellectual disability. Giving this lecture over time led one participant to suggest expanding her lecture to include new topics. The most experienced lecturers clearly described a gradual coping process making them more independent and introducing feelings of increased competence in the role of lecturer. These competence experiences also implied feelings of positive self-esteem.

Another source of competence experiences was the feedback provided during and after the lectures. The participants reported being told by the teacher present in the classroom that they were good at lecturing. The informants also said that they appreciated comments about the importance of the examples they gave from their everyday life with disabilities. One informant talked explicitly about the importance of receiving ‘praise from *an employer’* in the form of staff and students, and how this praise contributed to building feelings of competence. Praise from an employer was perceived as something special and was experienced differently from getting praise from parents or friends. The participant described how praise from the employer gave rise to ‘new feelings of competence’:

....*when I meet them, I get a pat on the back, and they say you are good... Then I feel: Yes, I have achieved what I wanted, finally.*

One of the participants described that she had not received sufficient feed-back from another teacher in the classroom and called for more support on how to organize her lecture. This participant seemed to imply that the lack of time to discuss her lecture with the present teacher evoked an insecurity that threatened her sense of mastery in the teaching situation.

Several of the informants had also acquired new computer skills during Covid, such as using Teams or Zoom to lecture. Another type of competence descriptions was when participants reported ‘flow-experiences’ in their role as lecturer, and they described how the relationship between level of challenge and level of competence was optimal. An example was this description of finishing a lecture:

*It’s like, now it is over, how boring! I do not even want to go home because it was such a thrill!*

Sometimes competence experiences were described as an outcome of ‘overcoming challenges’, for example when participants described still having some *‘butterflies in their stomach’* as they entered the classroom but still carried through. Some reported greater challenges than others in terms of acquiring this type of social competence. One informant described what it had been like to cope with this job assignment even though she had anxiety about large gatherings of people and sometimes found it difficult to go shopping. At university, however, she said she got to talk about *‘something that I am passionate about’*, and thus she learned ‘coping with performance pressure’. She shared what she had learned and how she had gained this social competence of lecturing in front of a group, saying:

*In a way, you must defy anxiety, and just face your fears.*

She conducted the lecture even though she said her *‘whole body was trembling’.* However, she said she had gradually learned to deal with this challenge, and she felt she had started to feel *‘kind of confident about it’*. Coping with the exposure to challenging social situations at university, seemed to have had a generalizing effect, and had provided her with new competences that contributed to reducing her anxiety in everyday life and made her dealing with the anxiety of going shopping much better.

Summing up, all the informants described the positive significance of acquiring new competences like ‘learning to lecture in front of a group’. They had learned to tell the story of their lives and to explore these stories together with other people. Adding ‘praise from *an employer’,* these experiences evoked ‘new feelings of competence’. Some participants also had powerful coping ‘experiences of flow’ and mastery in the classroom. Another essential source of competence experiences was ‘overcoming challenges’ like their own fears of mastering a complex social competence like giving a lecture.

### Experiences regarding self-determination

The main theme of self-determination evolved from sub-themes like ‘feeling influential in the classroom’, and describing ‘freedom to define their own Life story’ and ‘validation experiences in class’ making them ‘feel less controlled by others’ thus gaining the courage to become a ‘spokesperson for other disabled people’ so that some aspired to ‘influencing the system’ but also confronted the burden of ‘taking responsibility’ for the outcomes of exercising one’s self-determination.

Several informants emphasized the experience of ‘feeling influential in the classroom’. In their role as lecturers, they described an experience of gaining power and being empowered. This power involved an opportunity to define and describe what ‘life is really like out there’, as one of the informants put it. Most informants described having ‘validation experiences in class’ when lecturing about their lack of autonomy. They felt that they gained recognition for the injustice and discrimination they had experienced and ‘freedom to define their own Life story’. According to the informants, it was the feedback they received in the classroom that made them feel more confident in trusting their own experiences of disempowerment. Participants described how their perception of reality was validated and how that made them ‘feel less controlled by others’ and gave them a voice in defining reality. One participant described the effect of this kind of recognition when talking about his experiences with lack of autonomy and humiliating transgressions of his basic human rights:

*It is not something I have imagined; it is something that is not okay.*

Several participants saw lecturing as an opportunity to give future service providers a better understanding of how service recipients experience their lives with disabilities. Furthermore, they described an ambition to convey a better understanding of what kind of changes they want in the practices of service providers, especially related to autonomy and self-determination for individuals with intellectual disabilities. These autonomy experiences seemed to contribute a sense of meaning and purpose in their lives and as one informant maintained:

*I want disabled people to have the best possible experience. When you get a chance to speak for others with intellectual disabilities, you must do it.*

Another participant said that it was important to improve disabled people’s quality of life. Three participants described this intention of ‘influencing the system’ on behalf of themselves and others through lecturing at the university. One participant said, ‘I want to give them an aha-experience, bring the subject matter to life’. He wanted students to understand the depth and seriousness of his experiences. However, as he said, these experiences also applied to so many other people. He did not just take on the role as lecturer, but also the role as ‘spokesperson for other disabled people’. He saw this as *‘a social mission’.* The mission seemed to be about being heard in society in general and thus gaining more control and autonomy in life for himself and for others with intellectual disabilities.

Several participants described empowerment experiences related to the role of lecturer. One of the participants stated that the students ‘learn something from me’. Another said, ‘I see that they take notes and pay attention’. The participants described being in a new position where *they* had the knowledge and thus the power to set the agenda in class. Several of the informants used the term ‘power’ to describe what they experienced. One of the lecturers said she felt like a ‘pop star’ in her role as a lecturer. The students got in touch and wanted to talk to her about what she thought about various topics and about what kind of changes in service provision she saw as necessary. There was an experience of being admired and experiencing a ‘status boost’, signaling a feeling of being in control and making choices of their own, or as one participant expressed it:

*I enjoy myself and get to do what I think is important in life!*

The status boost was also experienced outside of the university when people expressed approval and respect related to their work assignment at the university. One participant said that acquaintances had admitted that: ‘daring to stand and give a lecture, is a brave thing to do’. Although the self-determination and autonomy related to lecturing at the university was empowering, some of the informants shared the experience that being in control and making their own choices could also have aspects that felt overwhelming. Autonomy in the form of ‘taking responsibility’ for criticism they had fronted or viewpoints they had shared as a spokesperson, sometimes resulted in mixed feelings and uncertainty, as one of them noted: *‘Can I vouch for what I have said?’* Another participant had made the choice of expressing her opinions in a direct manner, and afterwards wondered whether that maybe had not come across in the way that she had planned. She was worried about the responsibility she had taken on being a spokesperson and criticizing the lack of autonomy on behalf of others with intellectual disabilities, without having asked them about their opinions.

Exercising autonomy and expressing their own opinions as lecturers in the university also seemed to trigger responses outside the university. Some parents seemed to have opinions about the way the participants were exercising their autonomy in class. Not all parents were equally comfortable with the informants talking rather openly about their private life with an intellectual disability, sometimes including family members in their stories, and they sometimes tried to influence what was included in the lecture. At least one of the participants experienced negative reactions outside the family. She described meeting people outside the university who had become aware of her lecturing assignment and told her she was trying to be ‘omniscient’ or mocking her as ‘queen at the bottom of the heap’.

Summing up, most participants shared experiences of ‘feeling influential in the classroom’. They talked about how they ‘gained recognition’ when their perception of reality was listened to and validated. Having their experience of the world out there accepted, made them ‘feel less controlled by others’. These experiences gave some of them the courage to use their autonomy as lecturers ‘influencing the system’ and taking on a role of ‘spokesperson for other disabled people’. Nevertheless, when making choices for themselves in class as to what to say and how to influence future service providers, they also experienced the other side of self-determination namely the burden of ‘taking responsibility’ and feeling accountable for the criticism and the opinions they chose to share.

## Discussion

This study has focused on the experiences that six lecturers with intellectual disabilities had regarding lecturing assignments in a health and social education program. Participants were asked to describe their experiences with lecturing, what the job was about, how they felt about lecturing, and talking to students and being with other teachers, what they learned and what was challenging for them in exploring this work assignment. In the interviews, the participants described experiences touching on many topics. Nevertheless, the informants described mainly positive experiences with exploring these work assignments in the form well-being, inclusion, flow, coping and being in control. The factors described as relevant to the outcome of these work life experiences were organized into three overarching main themes. (1) Experiences regarding the establishment and maintaining of social relations. (2) Learning and coping experiences, and (3) Experiences regarding self-determination. When trying to contextualize these main themes in the fourth stage of the systematic text condensation, we realized that the main themes seemed rather familiar, because they so clearly alluded to the three basic needs identified in Deci and Ryan’s (27) self-determination theory (SDT): relatedness, autonomy, and competence. In the discussion, we will therefore focus on the participants’ experiences in relation to these three needs.

Several studies have emphasized the importance of *paying attention to the need satisfaction* of workers with intellectual disabilities (6, 28–30). This urge to pay attention to need satisfaction calls for further exploration of the interaction between the needs of people with intellectual disabilities and their working environment and their experiences with these interaction processes. One of the dominant theories explaining need satisfaction and internal motivation is self-determination theory (SDT). SDT is a macro-theory of human motivation which is applied in many different fields, including education and working life (27, 31–33). Deci and Ryan (34) focus on intrinsic motivation since people thus motivated experience more well-being and perform better compared to those who are motivated by extrinsic motivational factors. However, maintaining such intrinsic motivation requires the presence of supportive conditions because motivation can be disturbed by *unsupportive conditions in the surrounding environment*. Intrinsic motivation is based on the satisfaction of three basic needs according to SDT, namely relatedness, competence, and autonomy (27).

### Work experiences and work outcomes in the form of relatedness

Receiving emotional support from a work colleague was described as essential to feeling secure and establishing trust in their work environment. Being met in the foyer upon arrival, having someone to eat lunch with and share teaching experiences with, and receiving feedback from on their own performance, was described as one of the most important reasons for the overall feeling of inclusion that they experienced in the workplace. These descriptions appear closely related to the criteria for the concept of relatedness in self-determination theory. Relatedness is here defined by establishing affiliation and interaction in the form of caring for and respecting others, as well as being shown care and respect and thus feeling included (26).

The importance of this relatedness became especially evident when the participants described insecurity in talking to students, confusion when faced with uncomfortable questions, or other challenges in the classroom. Being able to seek emotional support from the other teacher and receiving respect and care from a colleague in such situations, was described by many as playing a crucial role in coping with classroom challenges, and a prerequisite for accessing flow experiences and well-being in the classroom. These descriptions also emerged as examples of how relationships and relatedness constitute a prerequisite for exercising one’s self-determination. The descriptions of the interaction with the other teacher in the classroom are strongly reminiscent of what Nonnemacher and Bambara (35) call supported decision-making. What the participants described can be understood as a form of ‘relational self-determination’ (35). Through emotional support and sometimes a bit of practical assistance, a permanently employed teacher helped the participants, when needed, to achieve the desired self-determination in the classroom. One of the participants described not receiving sufficient feedback on the organization and content of her lecture. This participant explicitly called for such support signaling a bit of insecurity in her relatedness to the students in class.

The participants’ descriptions of relatedness are particularly important because people with intellectual disabilities differ from the general population in terms of social participation. Drabløs (36) points out that for people with intellectual disabilities, the parental family plays a special role as their prime source of relatedness and that this does not change much over time. The importance of the parental family in their lives is partly because people with intellectual disabilities develop fewer friendships and have less access to various social arenas than others. It is often the care staff in the home who are described as playing a key role in their social network (36). The Norwegian Parliament, however, has addressed the importance of the workplace as a social arena for *all citizens*. In a report to Parliament, we can read that ‘Working life is one of the most central gateways to community and participation in society’ (37, p. 43). This statement provides an important context for understanding the significance of the descriptions of relatedness provided by the participants in our study. Even though the participants in the study did not have a permanent daily job at the institute for which they lectured, several of them felt that they had become affiliated with one or more new social arenas. Their descriptions of relatedness involved positive self-esteem, being affiliated with colleagues and students outside their home and family and having positive feed-back and social experiences remembered months and years later.

At the same time, it turned out that the relatedness they experienced in these working assignments also came with a certain cost. The participants had experienced relational challenges related to *self-disclosures* when they based the lecture on their own experiences. Self-disclosure is a process in which people involve others in personal information about themselves (38). The degree of self-disclosure is particularly important early on in a relationship because it has an impact on whether people wish to continue the interaction and develop a relationship (39). These challenges became evident in the descriptions of the interaction the participants had with the students during lecturing. Self-disclosures aroused the students’ interest and the students actively searched for such self-disclosures through the questions they asked. However, for the self-disclosures to stimulate relatedness in social interaction, the relationship must adapt to such disclosures and the situation must be perceived as being appropriate for such a disclosure (40). The person disclosing personal information must feel comfortable doing so to the recipient in question, and the recipient must be perceived as being trustworthy or likable. The self-disclosure must be perceived as meaningful to the persons and in the situation in question (41). The participants in our study generally expressed confidence that the situation was appropriate for a certain degree of self-disclosure. To a large extent, they perceived the recipients as people they could trust, even though some had initial doubts. The students’ reception of the information was of such a nature that it elicited trust and feelings of meaning because the reception was perceived as supportive and validating, and thus contributed to experiences of inclusion and relatedness.

Several participants stressed one type of self-disclosure contributing to their sense of relatedness in the workplace. When their experiences of violations and discrimination were given attention and accepted by the students in the classroom, they faced a feeling of having their perception of reality validated. Some of the things they had experienced in relation to helpers/assistants ‘were not okay’. The acknowledgement by others in the classroom were experienced as supportive. The concept of relatedness in self-determination theory includes the need to receive and provide care, attention, and support (26). Deci and Ryan (26) argue that the type of support inherent in validating feedback can be identity-affirming and therefore contribute to the establishment of relatedness. This statement very accurately addresses the experiences that the informants described considering what inspired their sense of inclusion and contributed to their relatedness.

### Work experiences and work outcomes in the form of competence, learning and coping

The participants also described many different examples of learning and coping experiences in their role as lecturers in higher education, ranging from gaining new IT skills, preparing PowerPoint presentations, and developing different types of social skills, such as being able to speak in front of an audience or taking the role of a spokesperson in front of a whole group of people. According to Deci and Ryan (27), we have a basic need to master different activities, and experience the feeling of *competence* when we succeed. The feeling of competence in working life is also linked to recognition and feedback. In line with this competency definition, the participants emphasized the importance of positive feedback and encouragement from other teachers, students, and the local community. They described how progress at work like mastering new challenges and gaining competence as lecturers, evoked strong feelings of motivation and flow.

Many of the participants also expressed that they had experienced mastering a new type of competence in their role as lecturer. They described representing both themselves and other people with intellectual disabilities when lecturing. This role carried new aspects of status, influence, and responsibility that they had not encountered before. In the field of disability, the term self-advocacy is often used to refer to this type of experience. It is described as acting as a *spokesperson* on behalf of oneself, through as Kartovicky (42) describes it, one’s ability to see one’s own resources and weaknesses and, based on this, being able to formulate personal goals, being self-assertive and making decisions. She further emphasizes that to become an advocate, both for themselves and others, disabled people must be given the opportunity to explore and get to know themselves to achieve a good understanding of the disability’s impact on their ability to cope, and an understanding of the adaptations they need to compensate for their challenges (42). It is precisely this kind of exploration of their own experiences, strengths, and limitations that the participants in our study described encountering in their conversations and interactions with students and teachers at their workplace.

Mastering the role of *spokesperson* was a demanding learning process for several, but at the same time it resulted in experiences of *progress*. All the participants described a sense of an increasing ability to cope in their role as lecturers. These coping experiences are particularly interesting considering that *progress* in work has been shown to be associated with well-being and high motivation, more often than any other workplace event (43) and is therefore an essential type of competency experience. In this context, it is important to remember that these coping experiences from ordinary working life only happened once or twice a year. Nevertheless, several of the participants described that these competency experiences had major positive consequences for their motivation to proceed with the work assignments at university and to involve themselves in new social arenas.

### Work experiences and work outcomes in the form of autonomy

Regarding experiences of autonomy, defined by Deci and Ryan (27) as involving the freedom of choice and the feeling of acting out of free will, many of the participants described how they themselves had the most influence over what they were going to say in class, and therefore they generally experienced a high degree of freedom in the lecturing situation. Several of the informants used the term ‘power’ to describe what they experienced. Power in this context involved the possibility to independently describe life with intellectual disability and the power to publicly criticise the lack of autonomy within the professional care they received. But it also involved the experience of having power when students found the lecturing interesting and gave the participants their undivided attention in the classroom. In fact, this experience of power was so strong in some of the informants that it was described as a sense of flow, almost a little ‘intoxicating’, in the sense that time flew by, and work and hobby became one, just as Csikszentmihalyi (44) describes in his Theory of flow.

Nevertheless, the participants accepted the rules of working life and found their autonomy within this framework, as Ravn [(45), p. 57] defines self-determination in working life. Some described rules regarding what one can say from the lectern, for example when it comes to swearing or getting too carried away by one’s emotions, as something they understood and saw the sense in. These rules were also something they had in common with all the other teachers and partly the students, a fact that also reminded them of being in a socially valued role and included in a working team. Just as in the study carried out by Taylor et al. (46), it is freedom *within* this framework that is described as motivating and contributing to work motivation and well-being at work.

The participants also described the power in discovering that they were not alone in their criticism. The audience in the classroom openly expressed that the participants’ rights *had* been violated in several contexts. These experiences were also described as promoting autonomy because the participants received confirmation that they could trust their own thoughts and experiences. The participants’ descriptions clearly showed how the need for autonomy holds a special position, as Deci and Ryan (26) claim, and how a lack of self-determination will disrupt the possibility of experiencing competence and relatedness (32). It is the experience of presenting their *self-determined* narratives about their own lives that the participants describe as the basis for both the sense of relatedness that occurred in the classroom and the coping experience they had. However, the participants clearly stated that the prerequisite for this coping experience was the relational self-determination they achieved through the support of the other teacher being in the classroom. Although the other teacher most of the time was rarely drawn into the interaction with the students, their presence was described as a prerequisite for the experience of autonomy. On the rare occasions when questions from students became too private or difficult, the other teacher was essential for maintaining the participants’ autonomy.

## Limitations

We used purposeful sampling aiming to interview participants with intellectual disabilities who could give information about how they experienced lecturing in this program. Based on this criterion sampling, we interviewed *all the lecturers with intellectual disabilities* involved in this health and social education program. Although this constituted a small sample of people with a relatively rare experience of lecturing in higher education, we found it relevant to interview them because few such studies can be found *describing the experiences of people with intellectual disabilities in this type of involvement.* There are evident limitations to a study based on the experiences of six lecturers. This sample represents a small population because very few individuals have this type of experience and findings cannot be generalized to large groups.

Furthermore, most of these participants had other jobs within both Permanently Adapted Work (PAW) and Permanently Adapted Work in Ordinary Companies (PAWO). In their usual jobs, our participants required very different levels of support. In other words, the in-group variation in our sample was considerable. Nevertheless, if we are to understand important aspects of facilitating the employment of individuals with intellectual disabilities, their experiences in varied working contexts must be studied and their experiences with varied requirements for support must be described. Qualitative research is defined as ‘the study of the nature of phenomena’, including ‘their quality, different manifestations, the context in which they appear or the perspectives from which they can be perceived’ (47). Thus, our contribution is to describe one such manifestation and the context in which it appeared.

Furthermore, the work experiences studied here were short-lived, in that they lasted about 2 h each time and happened once or twice a year. We thus found it important to investigate if the lecturers with intellectual disabilities experienced these limited work assignments as having any kind of impact on their lives in general.

## Conclusion

This study came about due to our interest in the practice of hiring people with intellectual disabilities to lecture at university in the capacity of being ‘experts on their own lives’. As observed in the introduction, prior studies on work inclusion of people with intellectual disabilities have primarily focused on assessing *the benefits of having a job,* and less on the processes and outcomes of such work experiences. In the present study, we have obtained rich descriptions of challenges and well-being in the lecturer role and the factors contributing to these experiences. According to the informants, their work motivation and well-being at work depended on the fulfilment of the three basic needs of belonging, competence, and autonomy. Fulfilling these three needs are theorized by Deci and Ryan (27) to be the prerequisites of inner motivation and well-being at work and in general. Testing self-determination theory empirically, Battaglia et al. (48) found that the three basic needs of belonging, competence, and autonomy increased employee satisfaction. The results of our study indicate that individuals with intellectual disabilities lecturing at the university focused on the same basic needs as individuals without intellectual disabilities, when describing the processes and outcomes found essential to their motivation and well-being at work.

## Data availability statement

The datasets presented in this article are not readily available because due to privacy concerns. Requests to access the datasets should be directed to rolf.wynn@gmail.com.

## Ethics statement

The study was reviewed and approved by the Norwegian Centre for Research Data (NSD project no. 454008). The participants have given both their oral and written consent to participate in the study.

## Author contributions

SM: Investigation, Writing – original draft, Writing – review & editing. LJ: Writing – original draft, Writing – review & editing. RW: Funding acquisition, Supervision, Writing – review & editing. GR: Supervision, Writing – original draft, Writing – review & editing.
